# A Multidisciplinary Assessment of ChatGPT’s Knowledge of Amyloidosis: Observational Study

**DOI:** 10.2196/53421

**Published:** 2024-04-19

**Authors:** Ryan C King, Jamil S Samaan, Yee Hui Yeo, Yuxin Peng, David C Kunkel, Ali A Habib, Roxana Ghashghaei

**Affiliations:** 1 Division of Cardiology Department of Medicine University of California, Irvine Medical Center Orange, CA United States; 2 Karsh Division of Gastroenterology and Hepatology Department of Medicine Cedars-Sinai Medical Center Los Angeles, CA United States; 3 School of Mathematics and Statistics Xi'an Jiaotong University Xi'an China; 4 GI Motility and Physiology Program Division of Gastroenterology University of California, San Diego La Jolla, CA United States; 5 Division of Neurology University of California, Irvine Medical Center Orange, CA United States

**Keywords:** amyloidosis, ChatGPT, large language models, cardiology, gastroenterology, neurology, artificial intelligence, multidisciplinary care, assessment, patient education, large language model, accuracy, reliability, accessibility, educational resources, dissemination, gastroenterologist, cardiologist, medical society, institution, institutions, Facebook, neurologist, reproducibility, amyloidosis-related

## Abstract

**Background:**

Amyloidosis, a rare multisystem condition, often requires complex, multidisciplinary care. Its low prevalence underscores the importance of efforts to ensure the availability of high-quality patient education materials for better outcomes. ChatGPT (OpenAI) is a large language model powered by artificial intelligence that offers a potential avenue for disseminating accurate, reliable, and accessible educational resources for both patients and providers. Its user-friendly interface, engaging conversational responses, and the capability for users to ask follow-up questions make it a promising future tool in delivering accurate and tailored information to patients.

**Objective:**

We performed a multidisciplinary assessment of the accuracy, reproducibility, and readability of ChatGPT in answering questions related to amyloidosis.

**Methods:**

In total, 98 amyloidosis questions related to cardiology, gastroenterology, and neurology were curated from medical societies, institutions, and amyloidosis Facebook support groups and inputted into ChatGPT-3.5 and ChatGPT-4. Cardiology- and gastroenterology-related responses were independently graded by a board-certified cardiologist and gastroenterologist, respectively, who specialize in amyloidosis. These 2 reviewers (RG and DCK) also graded general questions for which disagreements were resolved with discussion. Neurology-related responses were graded by a board-certified neurologist (AAH) who specializes in amyloidosis. Reviewers used the following grading scale: (1) comprehensive, (2) correct but inadequate, (3) some correct and some incorrect, and (4) completely incorrect. Questions were stratified by categories for further analysis. Reproducibility was assessed by inputting each question twice into each model. The readability of ChatGPT-4 responses was also evaluated using the *Textstat* library in Python (Python Software Foundation) and the *Textstat readability* package in R software (R Foundation for Statistical Computing).

**Results:**

ChatGPT-4 (n=98) provided 93 (95%) responses with accurate information, and 82 (84%) were comprehensive. ChatGPT-3.5 (n=83) provided 74 (89%) responses with accurate information, and 66 (79%) were comprehensive. When examined by question category, ChatGTP-4 and ChatGPT-3.5 provided 53 (95%) and 48 (86%) comprehensive responses, respectively, to “general questions” (n=56). When examined by subject, ChatGPT-4 and ChatGPT-3.5 performed best in response to cardiology questions (n=12) with both models producing 10 (83%) comprehensive responses. For gastroenterology (n=15), ChatGPT-4 received comprehensive grades for 9 (60%) responses, and ChatGPT-3.5 provided 8 (53%) responses. Overall, 96 of 98 (98%) responses for ChatGPT-4 and 73 of 83 (88%) for ChatGPT-3.5 were reproducible. The readability of ChatGPT-4’s responses ranged from 10th to beyond graduate US grade levels with an average of 15.5 (SD 1.9).

**Conclusions:**

Large language models are a promising tool for accurate and reliable health information for patients living with amyloidosis. However, ChatGPT’s responses exceeded the American Medical Association’s recommended fifth- to sixth-grade reading level. Future studies focusing on improving response accuracy and readability are warranted. Prior to widespread implementation, the technology’s limitations and ethical implications must be further explored to ensure patient safety and equitable implementation.

## Introduction

### Background

Amyloidosis is a rare, multisystem disease that comprises several subtypes including secondary amyloidosis, light chain amyloidosis, and ATTR (transthyretin amyloidosis), with the latter 2 being the most common but often underdiagnosed [1]. Light chain amyloidosis is diagnosed in 2500 to 5000 individuals annually in the United States, while the exact incidence of ATTR and secondary amyloidosis remains unknown due to challenges and delays in diagnosis stemming from a broad range of symptoms affecting multiple organ systems [2,3]. Diagnosing and caring for patients living with amyloidosis necessitate effective multidisciplinary collaboration between specialists in fields including but not limited to cardiology, gastroenterology, and neurology [4].

Due to amyloidosis being a rare disease, patients may be at risk for decreased health literacy regarding their condition. A notable scarcity of patient education materials (PEMs) exists for rare diseases compared to common ones, with one study showing nearly a 10-fold difference in the availability of PEMs related to rare diseases, which has been shown to adversely affect health outcomes [5]. According to the Centers for Disease Control and Prevention [6], improved health literacy could prevent up to 1 million hospitalizations annually and save US $25 billion in total health care costs.

ChatGPT (OpenAI), a large language model (LLM) powered by artificial intelligence released in late 2022, may be a powerful tool for improving the availability of accurate and readable information for rare and complex diseases like amyloidosis. Unlike traditional search engines, ChatGPT generates human-like text in a conversational format through an intuitive user interface. This is achieved with reinforcement learning from human feedback, wherein the model’s responses are refined through feedback loops to optimize responses [7]. With ongoing improvement and training using an extensive data set spanning diverse topics including medicine, ChatGPT’s accuracy and reliability in answering questions are expected to improve.

### Prior Work

Prior studies have demonstrated ChatGPT’s impressive accuracy and reliability in answering clinical questions across multiple medical specialties [8-10]. One study found the model’s generated responses were significantly higher in both quality and empathy compared to physicians when answering medical questions posted to social media, further bolstering the dynamic nature of this technology [11]. In March 2023, ChatGPT-4, the successor to ChatGPT-3.5, was released and has demonstrated superior performance in answering clinical questions across multiple fields of medicine [12-15]. In addition to accuracy and reliability, the readability of ChatGPT’s responses is an active area of investigation. Several studies related to ophthalmology and endocrinology have revealed that responses by ChatGPT-4 often exceed the fifth- to sixth-grade reading level recommended by the American Medical Association (AMA) [16-18]. While the literature examining LLM responses to clinical questions is growing, studies examining rare diseases are limited. Furthermore, there are currently no studies examining ChatGPT’s ability in answering questions related to amyloidosis.

### Aims of This Study

As with any emerging technology, rigorous evaluation of these models’ capabilities and limitations is essential to ensuring effective and safe implementation during their nascent stages before broad adoption by patients and providers*.* This study aims to build upon previous literature by using a multidisciplinary approach in assessing ChatGPT’s (1) accuracy in answering questions related to amyloidosis, particularly concerning cardiology, gastroenterology, and neurology; (2) reproducibility of responses; (3) readability; and (4) comparison of performance between ChatGPT-4 and ChatGPT-3.5.

## Methods

### Question Curation

A total of 98 amyloidosis-related questions were sourced from the frequently asked questions section of websites for professional medical societies and institutions. Questions from amyloidosis Facebook support groups were also incorporated to represent a more comprehensive patient perspective. Of these questions, 56 addressed general amyloidosis topics, while 42 were specific to cardiology (n=12), gastroenterology (n=15), and neurology (n=15). Each question was inputted twice into ChatGPT-4 (version updated on March 14, 2023) and ChatGPT-3.5 (version updated on February 9, 2023) except for neurology-related questions, which were only inputted into ChatGPT-4 due to reviewer availability. At the time of data collection, ChatGPT-4 required a paid monthly subscription. Furthermore, the models were without internet access, and their training data were limited to information prior to September 2021.

### Accuracy and Reproducibility

The accuracy of responses was assessed using the scale: (1) comprehensive, (2) correct but inadequate, (3) some correct and some incorrect, and (4) completely incorrect. Reproducibility was evaluated by categorizing each of the 2 responses of each question into those containing either no incorrect information (comprehensive and correct but inadequate) or those with incorrect information (some correct and some incorrect and completely incorrect). Questions that produced responses in different grading categories were deemed nonreproducible. Two independent reviewers (RG and DCK), board-certified in cardiology and gastroenterology with expertise in amyloidosis, assessed general amyloidosis questions and those of their respective specialties. Discrepancies in general question grading were resolved through discussion to reach a consensus. An additional reviewer (AAH), board-certified in neurology and specializing in amyloidosis, graded the neurology-specific responses for ChatGPT-4.

### Readability

The readability of ChatGPT-4’s responses was also assessed using the *Textstat* library in Python (Python Software Foundation) and the *Textstat readability* package in R software (R Foundation for Statistical Computing). The readability level was quantified either as a readability index or by using a predicted grade level, the latter indicating the US educational grade, at which the responses are comprehensible.

### Statistical Analysis

Categorical variables were presented as counts and percentages, while continuous variables were presented as means and SDs. Bivariate analysis consisted of Fisher exact test for categorical variables. Microsoft Excel (version 16.68; Microsoft Corp) was used for all statistical analysis.

### Ethical Considerations

Since all responses and outputs from ChatGPT were publicly available, approval from the institutional review board was not sought, and no informed consent was required.

## Results

In this study, ChatGPT’s responses were predominantly correct and also comprehensive (Table 1). Specifically, ChatGPT-4 (n=98) provided correct answers in 93 (95%) instances, with a notable 82 (84%) being graded as comprehensive. ChatGPT-3.5 (n=83) also performed well, delivering correct answers for 74 (89%) cases and comprehensive responses in 66 (79%) cases.

**Table 1:** Accuracy of responses by ChatGPT-3.5 and ChatGPT-4 to amyloidosis-related questions stratified by question subgroup.

Question subgroup		Responses, n (%)		
		ChatGPT-3.5		ChatGPT-4
**Overall** **(n=83 for** **Chat** **GPT-3.5 and n=98 for** **Chat** **GPT-4)**				
	Comprehensive	66 (79)	82 (84)	
	Correct but inadequate	8 (10)	11 (11)	
	Some correct and some incorrect	8 (10)	5 (5)	
	Completely incorrect	1 (1)	0 (0)	
**General questions (n=56)**				
	Comprehensive	48 (86)	53 (95)	
	Correct but inadequate	4 (7)	3 (5)	
	Some correct and some incorrect	4 (7)	0 (0)	
	Completely incorrect	0 (0)	0 (0)	
**Cardiology questions (n=12)**				
	Comprehensive	10 (83)	10 (83)	
	Correct but inadequate	0 (0)	2 (17)	
	Some correct and some incorrect	2 (17)	0 (0)	
	Completely incorrect	0 (0)	0 (0)	
**Gastroenterology questions (n=15)**				
	Comprehensive	8 (53)	9 (60)	
	Correct but inadequate	4 (27)	3 (20)	
	Some correct and some incorrect	2 (13)	3 (20)	
	Completely incorrect	1 (7)	0 (0)	
**Neurology questions (n=15)**				
	Comprehensive	—^a^	10 (67)	
	Correct but inadequate	—	3 (20)	
	Some correct and some incorrect	—	2 (13)	
	Completely incorrect	—	0 (0)	

^a^Not available.

When stratified by question category, both ChatGPT-4 and ChatGPT-3.5 excelled in general topics (n=56), where 53 (95%) and 48 (86%) of their responses, respectively, were comprehensive, though this difference was not statistically significant (*P*=.12). For cardiology, ChatGPT-4 was particularly accurate, correctly answering all 12 questions compared to ChatGPT-3.5’s 10 (83%) responses (*P*=.48). In gastroenterology (n=15), both models produced correct responses for 80% (n=12) of questions. However, their comprehensiveness varied slightly with ChatGPT-3.5 at 8 (53%) and ChatGPT-4 at 9 (60%). In neurology (n=15), ChatGPT-4’s responses were graded as comprehensive for 10 (67%).

Overall, ChatGPT-3.5 and ChatGPT-4 generated incorrect information in 9 of 83 (11%) and 5 of 98 (5%) responses, respectively. Notably, ChatGPT-3.5 produced 1 “completely incorrect” response regarding amyloidosis treatment of the gastrointestinal tract, involving the recommendation of probiotics and digestive enzymes (Multimedia Appendix 1). An example of a “some correct and some incorrect” response from ChatGPT-3.5 related to the management of atrial fibrillation in patients with amyloidosis. The model correctly described similar rate control and anticoagulation strategies for patients with amyloidosis having atrial fibrillation compared to those without amyloidosis but understated the prevalence of atrial fibrillation in ATTR. ChatGPT-4, on the other hand, did not produce any completely incorrect responses but did provide a response categorized as “correct but inadequate” by omitting autonomic symptoms in amyloidosis-related neuropathy. Regarding reproducibility, ChatGPT-4 showed a higher rate of 96 of 98 (98%) reproducible responses compared to 73 of 83 (88%) for ChatGPT-3.5 (Table 2).

**Table 2:** Reproducibility of responses by ChatGPT-3.5 and ChatGPT-4 to amyloidosis-related questions categorized by question subgroup.

Question subgroup	Responses, n (%)	
	ChatGPT-3.5	ChatGPT-4
Overall (n=83 for ChatGPT-3.5 and n=98 for ChatGPT-4)	73 (88)	96 (98)
General (n=56)	49 (88)	55 (98)
Cardiology (n=12)	10 (83)	12 (100)
Gastroenterology (n=15)	14 (93)	15 (100)
Neurology (n=15)	—^a^	14 (93)

^a^Not available.

In terms of readability, ChatGPT-4’s responses varied but were consistently well above the AMA’s recommended fifth- to sixth-grade reading level. The Flesch-Kincaid Grade Level scale rated them between a high school sophomore and a graduate level, averaging at a college level (mean 15.5, SD 1.9; range 10.3-21.7; Table 3). The Flesch Reading Ease scores, on a scale of 0 to 100, averaged at 23.3 (SD 9.4), indicating a college graduate level of complexity. Additional readability metrics showed a broad range of scores, all with similar advanced reading levels: Simple Measure of Gobbledygook (range 12.8-20.2), Gunning Fog Index (range 14.3-24.2), Coleman-Liau Index (range 10.5-18.3), Automated Readability Index (range 9.9-24.3), FORCAST Grade Level (range 10.3-13.4), and Powers Sumner Kearl Grade (range 6.8-9.4).

**Table 3:** Readability of responses by ChatGPT-4 to amyloidosis-related questions.

Readability metric	Score, mean (SD)	Range
Flesch Reading Ease	23.3 (9.4)	5.6-47.9
Flesch-Kincaid Grade Level	15.5 (1.9)	10.3-21.7
Simple Measure of Gobbledygook	16.7 (1.6)	12.8-20.2
Gunning Fog Index	19.1 (2.3)	14.3-24.2
Coleman-Liau Index	15.3 (1.4)	10.5-18.3
Automated Readability Index	15.6 (2.1)	9.9-24.3
FORCAST Grade Level	12.1 (0.53)	10.3-13.4
Powers Sumner Kearl Grade	8.2 (0.55)	6.8-9.4

## Discussion

### Principal Results

Literature examining ChatGPT’s knowledge regarding rare diseases, such as amyloidosis, is limited compared to that of more prevalent health conditions. In this study, we employed an interdisciplinary panel of amyloidosis experts from cardiology, gastroenterology, and neurology to evaluate the accuracy and reproducibility of ChatGPT-4’s and ChatGPT-3.5’s responses to amyloidosis-related questions. Furthermore, the readability of responses by ChatGPT-4 was examined. ChatGPT-4 and ChatGPT-3.5 produced comprehensive responses to 53 (95%) and 48 (86%) general questions, respectively. Incorrect information was found in 5 of 98 (5%) and 9 of 83 (11%) responses from ChatGPT-4 and ChatGPT-3.5, respectively (*P*=.17), with 1 of 83 (1%) ChatGPT-3.5 responses graded as completely incorrect. The models also provided high reproducibility in accuracy of responses overall, with ChatGPT-4 and ChatGPT-3.5 generating 96 of 98 (98%) and 73 of 83 (88%) reproducible responses, respectively. However, the readability of ChatGPT-4’s responses exceeded the AMA’s recommended fifth- to sixth-grade reading level for PEMs, with readability at a college reading level on average.

### Comparison With Prior Work

Previous studies have shown ChatGPT’s impressive knowledge when assessing both common and rare diseases. The model has displayed extensive knowledge regarding cardiovascular disease prevention [8]. In more intricate scenarios such as clinical vignettes describing atrial fibrillation, congenital heart disease, and heart failure, its answers were assessed as predominantly reliable, valuable for patients, and crucially, not hazardous. Interestingly, many of these responses were favored over those generated by a standard Google search [19]. Similar results have been shown in several studies involving gastrointestinal-related topics such as cirrhosis, hepatocellular carcinoma, and bariatric surgery [9,10], with ChatGPT-4 demonstrating a significant improvement in knowledge compared to ChatGPT-3.5 [12,15]. Mehnen et al [13] demonstrated superior diagnostic precision of rare diseases by ChatGPT-4 compared to ChatGPT-3.5 as well. Our results showed comparable overall accuracy and reproducibility to previous studies, with both models generating consistent and reliable information. Although not meeting the level of significance as seen in prior research, ChatGPT-4 did generate fewer responses with incorrect information than ChatGPT-3.5 in this study.

The superior performance of ChatGPT-4 in prior studies may stem from multiple factors inherent to the design of each model. ChatGPT-4 was trained on a larger body of information, potentially exposing the model to a wider range of medical information. ChatGPT-4 has been reported to possess more advanced reasoning capabilities, allowing the model to better formulate explanations tailored to the input provided. Finally, the training of ChatGPT-4 may have provided the model with an advantage [14].

### Limitations of ChatGPT

ChatGPT holds the potential to enhance clinical practice in the context of amyloidosis, but notable limitations exist. Chief among these is the undisclosed origin of ChatGPT’s primary training data set, paired with its inability to regularly provide citations for its responses. Directly referencing established medical sources would bolster its clinical credibility. Moreover, ChatGPT sometimes produces responses referred to as “hallucinations,” which are confident sounding, yet completely incorrect answers. The data set’s scope is further limited to information prior to September 2021 [7]. The quality of responses generated by ChatGPT is affected by the nature of the prompts inputted by the user. Prompt engineering has been shown to significantly alter the models’ output both in quality and comprehensiveness. Future studies would benefit from including the testing of different prompts and their effect on response output in the context of amyloidosis. Furthermore, concerted efforts in increasing patient and provider knowledge regarding prompt engineering may better facilitate the future effective use of these models. This study highlights the need for improvements in response readability to ensure equitable use of this technology across all patient populations. Similarly, other studies involving hypothyroidism in the setting of pregnancy and retinal surgery have also noted ChatGPT to produce information at a college reading level and beyond [17,18]. Furthermore, the majority of studies in the literature have examined the model’s performance in English, with a limited body of literature examining non-English languages [20-22]. More studies are needed to ensure the optimization of model performance across a wide range of languages.

### Ethical Implications

Beyond model-specific challenges, ethical issues remain unresolved. Potential biases introduced during training could skew user outputs. Clinical research bias, such as the overrepresentation of White populations [23], might also persist within the model. There is a growing body of literature examining implicit bias in responses from LLMs with conflicting results [24-26]. Equitable access is another concern; lower socioeconomic groups might face barriers in accessing such technology due to hardware and internet constraints. Privacy is a further point of contention, though OpenAI’s option to disable chat history storage addresses some concerns [27]. Regulatory oversight, as suggested by the Food and Drug Administration, is paramount. The proposed regulation would align artificial intelligence health care tools with medical device standards, emphasizing repeated validation and testing at each stage of development [28]. Additionally, physician panels should advise technical developers, ensuring patient safety and prioritizing equitable, outcome-driven patient care.

### Strengths and Limitations of This Study

This study’s strengths include being among the first in using a multidisciplinary approach to evaluate ChatGPT’s knowledge of amyloidosis. This holistic approach enabled a thorough assessment of ChatGPT’s abilities in addressing clinical queries related to amyloidosis, a rare disease necessitating advancements in health education, diagnostics, and management for improved patient outcomes. However, this study is not without its limitations. We relied on a single physician reviewer for specialty-specific responses, which is subjective and prone to bias. Research could bolster validity by engaging multiple reviewers within each specialty to minimize the potential for subjective bias. It would also be beneficial to include physicians specializing in hematology, oncology, and nephrology as reviewers due to their integral involvement in caring for patients with amyloidosis. Furthermore, we recommend including patients and all members of the health care team when reviewing the quality of responses. While we took a systematic approach when curating questions, our list may not comprehensively represent all potential patient questions related to amyloidosis.

### Conclusions

ChatGPT delivered accurate and reliable responses to amyloidosis-related questions across general and specialty-specific questions. ChatGPT has the potential to serve as a supplemental tool in disseminating vital health education to patients in the future. However, the presence of some incorrect responses underscores the necessity of continued improvements and fine-tuning of future iterations prior to incorporation into clinical practice. Furthermore, improvement in the readability of responses is essential to ensuring equal access to this technology by all patients. We advocate for the use of this technology as an adjunct and not a replacement to care and advice provided by licensed health care professionals. In its current state, there are also limitations and ethical concerns that need to be resolved before the technology may be widely implemented in health care in a safe and equitable manner.

## Appendix

Multimedia Appendix 1 Examples of prompts with corresponding ChatGPT responses and reviewer accuracy grades.

## Abbreviations

AMA: American Medical Association
ATTR: transthyretin amyloidosis
LLM: large language model
PEM: patient education material

## Data Availability

The data sets generated and analyzed during this study are available from the corresponding author on reasonable request.
