# Supplementary material for: A Multidisciplinary Assessment of ChatGPT’s Knowledge of Amyloidosis: Observational Study
Source: JMIR Cardio. 2024 Apr 19;8:e53421. doi: 10.2196/53421 (PMC11069089; doi:10.2196/53421)
Supplement: Multimedia Appendix 1 [file cardio_v8i1e53421_app1.docx]

| Prompt | GPT response | GPT version; Grade (1-4) | Reviewer comment |
| --- | --- | --- | --- |
| What is serum neurofilament light chain in relation to amyloidosis? | Serum neurofilament light chain (sNfL) is a marker of neuroaxonal damage. Neurofilaments are structural proteins in neurons, and the presence of neurofilament light chain in the bloodstream can indicate nerve cell damage. Increases in sNfL concentrations in the blood and CSF (cerebrospinal fluid) are observed in many neurological conditions, including multiple sclerosis, Alzheimer's disease, and amyotrophic lateral sclerosis (ALS).  Amyloidosis is a group of diseases in which abnormal proteins called amyloids build up in tissues and organs. Amyloid proteins can be deposited in a localized area and may not be harmful, or they can cause serious damage by disrupting the normal function of one or more organs. There are different types of amyloidosis, including those that affect the nervous system, such as familial amyloid polyneuropathy.  Amyloidosis that affects the nervous system could potentially cause neuroaxonal damage, leading to increased levels of sNfL. However, as of my knowledge cutoff in September 2021, there's limited research explicitly connecting sNfL levels to amyloidosis. Nonetheless, in theory, it could serve as a useful biomarker for monitoring the progression or response to treatment in neurological types of amyloidosis.  If there's been more recent research linking sNfL and amyloidosis since my last training data in September 2021, you would need to check the latest studies or consult a healthcare professional for the most up-to-date information. | GPT-4; 1, comprehensive | - |
| What is sensorimotor polyneuropathy caused by amyloidosis? | Sensorimotor polyneuropathy caused by amyloidosis refers to a specific type of nerve damage that occurs due to the deposition of abnormal protein fibers, called amyloid, in and around the peripheral nerves. This can lead to a wide range of symptoms affecting both sensory and motor functions.  Amyloidosis is a group of diseases characterized by the accumulation of amyloid proteins in various organs and tissues throughout the body. These proteins can accumulate in different types of cells, causing dysfunction and eventually leading to organ failure.  In the case of sensorimotor polyneuropathy, amyloid proteins primarily deposit in the peripheral nerves, which are responsible for transmitting sensory information to the central nervous system and controlling muscle movement. This can result in a range of symptoms, including:  Sensory symptoms: Numbness, tingling, or burning sensations in the extremities, especially the hands and feet. This can lead to a reduced ability to feel pain, temperature changes, and vibration, as well as an increased risk of injury.  Motor symptoms: Muscle weakness, cramping, and atrophy, which may lead to difficulty walking, loss of fine motor skills, or muscle wasting.  Sensorimotor polyneuropathy caused by amyloidosis is typically a progressive condition, and the severity of the symptoms tends to increase over time. Treatment options may include managing the underlying cause of the amyloidosis, such as treating plasma cell disorders or addressing inflammatory conditions, as well as symptom management, including pain relief and physical therapy. In some cases, medications, such as immunosuppressive drugs, or procedures, like stem cell transplantation, may be used to slow the progression of the disease. | GPT-4; 2, correct but inadequate | The first response does not mention autonomic involvement which is often the flag that makes amyloid neuropathy higher in the differential when assessing patients. |
| How common is atrial fibrillation in amyloidosis and how is it treated? | Atrial fibrillation (AF) is a common cardiac manifestation of amyloidosis. The incidence of AF in amyloidosis patients varies depending on the type of amyloidosis and the severity of cardiac involvement. In AL amyloidosis (amyloid light-chain), AF occurs in up to 30-40% of patients, while in ATTR amyloidosis (transthyretin-related), AF is less common but still occurs in around 10-15% of patients.  The treatment of AF in amyloidosis patients is generally similar to that in patients without amyloidosis. However, because of the underlying amyloidosis and associated cardiac involvement, the management of AF in these patients may be more challenging.  The first-line treatment for AF is typically anticoagulation with medications such as warfarin or direct oral anticoagulants (DOACs) to reduce the risk of stroke. In patients with amyloidosis, careful consideration is needed to assess the risk of bleeding due to the potential for amyloid deposition in blood vessels.  In addition to anticoagulation, rate control or rhythm control strategies may be used to manage AF symptoms. Rate control involves using medications such as beta-blockers or calcium channel blockers to slow the heart rate, while rhythm control involves using medications such as antiarrhythmic drugs or cardioversion (electric shock to restore normal rhythm) to restore and maintain normal heart rhythm.  In some cases, catheter ablation may be considered as a treatment option for AF in amyloidosis patients. However, this procedure may be more challenging in patients with amyloidosis due to the potential for amyloid deposition in the heart tissue.  Overall, the treatment of AF in amyloidosis patients requires a careful assessment of the risks and benefits of different treatment options and close collaboration between cardiologists and amyloidosis specialists. | GPT-3.5;  3, some correct and some incorrect | Afib is very common in ATTR. |
| Are there any supplements that can improve gastrointestinal symptoms from amyloidosis? | Amyloidosis is a rare disorder characterized by the buildup of abnormal proteins in different tissues and organs, including the gastrointestinal (GI) tract. The symptoms of GI amyloidosis can be variable and may include diarrhea, constipation, nausea, vomiting, abdominal pain, and weight loss.  While there are no supplements that can cure amyloidosis, there may be some supplements that can help improve GI symptoms associated with the condition. However, it is important to note that the use of supplements for medical purposes should always be discussed with a healthcare provider before starting.  Some supplements that have been suggested to improve GI symptoms in amyloidosis include:  Probiotics: These are beneficial bacteria that can help restore the balance of gut flora and improve digestion. Some studies have shown that probiotics may help reduce diarrhea, bloating, and abdominal discomfort in people with amyloidosis.  Digestive enzymes: These supplements contain enzymes that help break down food and aid in digestion. They may be helpful for people with amyloidosis who have difficulty digesting certain foods, such as fats or carbohydrates.  Fiber supplements: These supplements can help regulate bowel movements and improve stool consistency. However, people with amyloidosis should be careful with fiber supplements, as they may worsen diarrhea or other GI symptoms.  Vitamin and mineral supplements: People with amyloidosis may be at risk of malnutrition due to difficulty absorbing nutrients from food. Supplements containing vitamins and minerals, such as iron, calcium, and vitamin B12, may help address deficiencies and improve overall health.  It is important to note that supplements should never be used as a substitute for medical treatment, and that they can interact with other medications or cause side effects. Always speak to a healthcare provider before taking any supplements or making changes to your diet or medication regimen. | GPT-3.5;  4, completely incorrect | Probiotics should not be suggested for amyloidosis; digestive enzymes should not be suggested for amyloidosis; vitamins only needed if deficiencies have been identified. |
